# Radiofrequency Ablation for Early-Stage Nonsmall Cell Lung Cancer

**DOI:** 10.1155/2014/152087

**Published:** 2014-06-03

**Authors:** Takao Hiraki, Hideo Gobara, Toshihiro Iguchi, Hiroyasu Fujiwara, Yusuke Matsui, Susumu Kanazawa

**Affiliations:** Department of Radiology, Okayama University Medical School, 2-5-1 Shikatacho, Okayama 700-8558, Japan

## Abstract

This review examines studies of radiofrequency ablation (RFA) of nonsmall cell lung cancer (NSCLC) and discusses the role of RFA in treatment of early-stage NSCLC. RFA is usually performed under local anesthesia with computed tomography guidance. RFA-associated mortality, while being rare, can result from pulmonary events. RFA causes pneumothorax in up to 63% of cases, although pneumothorax requiring chest drainage occurs in less than 15% of procedures. Other severe complications are rare. After RFA of stage I NSCLC, 31–42% of patients show local progression. The 1-, 2-, 3-, and 5-year overall survival rates after RFA of stage I NSCLC were 78% to 100%, 53% to 86%, 36% to 88%, and 25% to 61%, respectively. The median survival time ranged from 29 to 67 months. The 1-, 2-, and 3-year cancer-specific survival rates after RFA of stage I NSCLC were 89% to 100%, 92% to 93%, and 59% to 88%, respectively. RFA has a higher local failure rate than sublobar resection and stereotactic body radiation therapy (SBRT). Therefore, RFA may currently be reserved for early-stage NSCLC patients who are unfit for sublobar resection or SBRT. Various technologies are being developed to improve clinical outcomes of RFA for early-stage NSCLC.

## 1. Introduction

Primary lung cancer is the most common cause of death due to cancer worldwide. If untreated, patients with primary lung cancer have a poor prognosis. Vrdoljak et al. [1] studied 19 patients with untreated clinical stage IB nonsmall cell lung cancer (NSCLC) and found that these patients had a mean survival time of 17 months and a 2-year survival rate of 20%. Another study by McGarry et al. [2] reported that 49 patients with untreated stage I or II cancer had a mean survival time of 14 months, with 53% of patients dying due to cancer.

The suggested first-line treatment for early-stage NSCLC is surgical resection. Although some surgeons believe that sublobar resection is effective for the treatment of localized cancer, lobectomies are still considered to be the gold standard because of a large randomized controlled trial that demonstrated that lobectomy was superior to limited resection in terms of both patient survival and locoregional recurrence in patients with T1N0 NSCLC [3]. Unfortunately, some patients are considered inoperable. Bach et al. [4] estimated that more than 20% of patients with early-stage lung cancer did not undergo surgery. These patients are traditionally treated with conventional external beam radiation therapy. A meta-analysis of stage I NSCLC patients treated with conventional external beam radiation therapy found that the mean overall survival and cause-specific survival rates of these patients at 3 years were 34% and 39%, respectively [5]. The survival outcomes associated with conventional external beam radiation are unsatisfactory; therefore, many studies have focused on various alternative modalities. Radiofrequency ablation (RFA) has received considerable attention as local therapy, mainly for hepatic cancer. The favorable outcomes obtained by RFA of hepatic cancer have encouraged the application of this technique to lung cancer.

Dupuy et al. [6] reported the first clinical use of RFA to treat lung cancer in 2000. Since then, RFA has been commonly used as a treatment for lung cancer. The United States Food and Drug Administration has approved RFA for the treatment of primary and metastatic tumors in soft tissue, including the lungs. Because the thermal and electrical conductivity of air are low, the effects of RFA on the lungs may be tissue-specific. Accordingly, studies have demonstrated that a given quantity of radiofrequency current ablates a larger volume of tumor in the lungs than in subcutaneous tissues or kidneys [7]. Nguyen et al. [8] performed an “ablate and resect study” that included 8 patients with clinical stage I or II NSCLC who were treated with RFA at the same time as they underwent a thoracotomy for surgical resection. Histological examination showed that 3 of the 8 tumors were completely ablated. All completely treated tumors were <2 cm in diameter. Ambrogi et al. [9] also performed an “ablate and resect study.” They confirmed histologically complete necrosis in 6 of 9 peripheral stage I or II NSCLC tumors. These results indicate that RFA shows potential as a treatment option for early-stage NSCLC. In this paper, we review the published literature for reports of outcomes of patients with early-stage NSCLC treated with RFA.

## 2. Review of Studies on RFA of NSCLC

A review of the literature was conducted by searching the PubMed database. The results were limited to studies published in English. The search was performed on February 24, 2014, using the keywords “nonsmall cell lung cancer” and “radiofrequency ablation.” The list of all electronically identified articles was then manually examined to distinguish potentially relevant studies. We selected human clinical studies on the efficacy of RFA in NSCLC and excluded animal experiments, case reports, and reviews. Preliminary clinical studies with small populations and studies that did not provide adequate survival data were also excluded. Moreover, studies that included patients treated with the combination of RFA and radiation were also excluded. All relevant articles were subsequently evaluated.

There were 14 relevant studies [10–23] from 4 institutes in the United States (US) [11–13, 16, 17, 21, 22], 4 institutes in Asia (Japan, South Korea, and China) [10, 14, 18, 19, 23], 1 institute in Europe (Italy) [20], and 1 multicenter trial from the US, Europe, and Australia [15]. The clinical results of RFA for the patients with NSCLC in the relevant studies are summarized in Table 1. There were several reports that included mixed populations comprising both primary and metastatic lung cancer patients [10, 13, 15, 19]; for these reports, we attempted to extract data that was only from NSCLC patients.

The majority of the relevant studies had a population size of 50 patients or less [10–12, 14–18, 22, 23], with the largest population being a Chinese study with 237 patients [19]. The median or mean patient age was usually 70 to 76 years [11, 14, 16–18, 20–23]. Many of the studies primarily involved patients with relatively small tumors; the median or mean sizes tended to be 2.0 to 3.0 cm [11–16, 18, 20, 22]. Thus, these studies mainly included patients with stage I, particularly stage IA, cancer. The histological type of most of the tumors was squamous cell carcinoma or adenocarcinoma, including bronchoalveolar carcinoma. The relevant studies were based on information obtained during short- or mid-term follow-ups, with all reported median or mean follow-up periods of shorter than 5 years [10–12, 14, 16, 18, 20–23]. Thus, these studies lacked long-term survival data.

RFA was usually performed on inpatients, with median or mean hospital stays of up to 5 days [11, 12, 14, 17, 19, 20]. The vast majority of procedures were performed using local anesthesia and under computed tomography (CT) guidance. Some of the procedures were performed via thoracotomy [11] or with ultrasound guidance for pleural-based tumors [20]. Although mortality was quite rare, it did occasionally occur due to acute respiratory distress [10], pulmonary embolus [11], and exacerbation of pulmonary fibrosis [13]. Pneumothorax was frequently associated with the procedures, with maximum of 63% of cases [12]. Pneumothorax requiring chest drainage occurred in 2% to 13% of the procedures in most of the studies [14–16, 18, 20, 22, 23] and accounted for most of the major complications. The other complications were pleural effusion [14, 16, 18, 20], hemothorax [16, 23], pneumonia or pneumonitis [11, 16, 18], neuropathy [16], bronchopleural fistula [16, 18], hemoptysis [16, 17, 20, 23], empyema [18], pain [20], chest wall hematoma [20], and pneumomediastinum [23].

The rate of local tumor progression after RFA of stage I NSCLC was similar among the studies: 31% to 42% [10, 12, 14, 16–18, 20, 22]. Ambrogi et al. [20] showed that the local control rate associated with RFA differed significantly between stages IA and IB cancer. The 1-, 2-, 3-, and 5-year overall survival rates after RFA of stage I NSCLC were 78% to 100% [10, 12–14, 16, 18, 20], 53% to 86% [10, 12–16, 18, 20], 36% to 88% [13, 14, 16–18, 20, 22], and 25% to 61% [13, 18, 20, 22], respectively. The median survival time ranged from 29 to 67 months [13, 16, 18, 20, 22, 23]. The 1-, 2-, and 3-year cancer-specific survival rates after RFA of stage I NSCLC were 89% to 100% [14, 18, 20], 92% to 93% [14, 15, 18], and 59% to 88% [14, 17, 18, 20], respectively. The 3-year disease-free survival rate after RFA of stage I NSCLC was 39% to 53% [16–18]. The 1- and 3-year overall survival rates after RFA of stage IA NSCLC were 84% to 95% [14, 18, 20, 21] and 71% to 84% [14, 18, 20, 21], respectively; the 1- and 3-year overall survival rates after RFA of stage IB NSCLC were 79% to 92% [14, 18, 21] and 50% or 67% [18, 21], respectively.

Data on RFA of early-stage NSCLC continue to accumulate. Most of the current information about the role of RFA in NSCLC comes from retrospective studies, so high-quality data is still lacking. The American College of Surgeons Oncology Group (ACOSOG) undertook a prospective phase II trial (Z4033) to assess the safety and efficacy of RFA in high-risk patients with stage IA NSCLC. The primary endpoint of their study was the 2-year survival rate. This study enrolled 54 patients up to July 2010, and survival data will be available soon.

## 3. Comparison of RFA and Other Local Therapies

### 3.1. Sublobar Resection

RFA may rival other local therapies such as sublobar resection and stereotactic body radiation therapy (SBRT). Recently, several studies have compared RFA with sublobar resection for the treatment of stage I NSCLC. Crabtree et al. [38] compared the selection criteria and short-term outcomes in 3 prospective clinical trials that used SBRT (Radiation Therapy Oncology Group [RTOG] trial 0236), sublobar resection (ACOSOG trial Z4032), and RFA (ACOSOG trial Z4033). The RFA trial included patients who were older and had more heavily impaired lung function. Mortality rates were not significantly different between the 3 modalities.

Kim et al. [39] retrospectively examined the outcomes of 8 patients with inoperable stage I NSCLC who were treated with RFA compared to 14 patients who were treated surgically. The rate of local recurrence was higher in the RFA group, but the 2 groups developed distant metastatic disease at the same frequency, and there was no difference in overall survival. Zemlyak et al. [17] reported the outcomes of 64 patients with stage I NSCLC who were deemed unsuitable for standard resection and were therefore treated with sublobar resection, RFA, or cryotherapy. Overall survival was similar among patients who received the 3 treatment modalities: 87% in the 25 patients who underwent surgery, 88% in the 12 patients treated with RFA, and 77% in the 27 patients treated with cryotherapy. Cancer-specific survival was also similar among the patients: 91% in the surgery group, 88% in the RFA group, and 90% in the cryotherapy group. There were trends toward higher recurrence in the RFA group and longer cancer-free survival in the surgical group, although these differences were not statistically significant.

Lee et al. [23] retrospectively compared the survival rate of 16 patients with stage I or II NSCLC treated with RFA to 13 patients treated with surgery. Although patient age was significantly higher in the RFA group, survival was not significantly different between patients who underwent RFA versus surgery (median survival: 28 months after RFA versus 34 months after surgery). Kwan et al. [40] used National Cancer Institute Surveillance, Epidemiology, and End Results-Medicare linked data to examine the survival of patients with early-stage NSCLC after thermal ablation and sublobar resection. The patients who were treated with thermal ablation were significantly older, had higher comorbidity index scores, and were more likely to have chronic obstructive pulmonary disease. Analyses of these 2 unmatched groups indicated significantly longer overall and cancer-specific survival for the patients who underwent sublobar resection. However, after propensity score matching, overall survival and cancer-specific survival were not significantly different between the 2 groups. These studies suggest that the increased frequency of local recurrence after RFA does not have a significant impact on overall or cancer-specific survival. This is probably because the patients who underwent RFA were older and tended to have substantial comorbidities, so they tended to die due to causes other than cancer recurrence. In contrast with the previously discussed studies, Alexander et al. [41] reported that 28 patients with stage I NSCLC treated with sublobar resection had significantly longer overall and cancer-specific survival and a lower risk of recurrence compared to 56 patients treated with RFA. It should be noted that this result was biased by the fact that the RFA group was significantly older than the surgical group.

### 3.2. SBRT

SBRT is associated with favorable local control and survival rates in patients with stage I NSCLC. The results of recent studies of SBRT for stage I NSCLC [24–37] are summarized in Table 2. This therapy did not result in mortality in the vast majority of the reported studies [24–27, 29, 30, 32–36]. However, 2 studies from the same group reported grade 5 toxicities in 7% and 9% of patients [28, 31]. Causes of death included pneumonia [28, 31], pericardial effusion [28], hemoptysis [28, 31], and respiratory failure [31]. Radiation pneumonitis sometimes occurs after SBRT; many of the studies reported that the incidence of grade 3 or greater pulmonary events was 5% or less [24–27, 29, 32, 34–36]. On the other hand, some reports showed that 10% to 30% of patients experienced grade 3 or 4 toxicities [28, 30, 31, 33].

Local recurrence was reported in up to 20% of patients [24–37] or 10% of patients or less in many of the studies of SBRT [24–26, 28, 30–35]. The 1-, 2-, 3- and 5-year overall survival rates were 80% to 95% [26, 27, 30, 35, 37], 55% to 75% [24, 26–28, 30, 32], 43% to 85% [27, 29–33, 35–37], and 25% to 70% [27, 29, 34–37], respectively. The median overall survival was 32 to 62 months [27, 28, 30, 31, 33, 35]. The 3-year and 5-year cancer-specific survival rates were 67% to 88% [27, 29–32] and 41% to 76% [27, 29, 34], respectively.

Sher et al. [42] performed a cost-effectiveness analysis of SBRT and RFA for medically inoperable, early-stage NSCLC. They found that SBRT was the more cost-effective treatment. On the basis of the studies discussed here, SBRT may provide more local efficacy but may slightly be more toxic than RFA and is associated with similar midterm survival outcomes as RFA.

Although SBRT rivals RFA, these two modalities may be, at the same time, complementary to each other. For example, RFA may be performed when performing SBRT seems hazardous, that is, when a tumor is located near the hilum, mediastinum, lung apex, and vertebral body and a tumor is located in the lower lobe in the patients with considerable respiratory motion. Considering that RFA seems to impair pulmonary function less than SBRT inducing radiation pneumonitis, RFA may be applied to the patients with severe pulmonary dysfunction. In contrast, considering more local efficacy by SBRT, larger tumors may be treated with SBRT.

## 4. Role of RFA in the Treatment of Early-Stage NSCLC

The American College of Chest Physicians (ACCP) guidelines for diagnosis and management of lung cancer, third edition [43], included RFA as a treatment option for peripheral tumors less than 3 cm in size in inoperable patients. RFA appears to result in a higher rate of local failure than sublobar resection and SBRT. Tumors greater than 3 cm in size are especially likely to recur locally after therapy. Although it is unclear how much this increased local failure impacts survival outcomes in old and high-risk patients, we suggest that sublobar resection and SBRT may be the preferred therapeutic options for patients with early-stage NSCLC who are unsuitable for lobectomy. Therefore, RFA may currently be reserved for patients who are unfit for sublobar resection or SBRT. This suggestion is in accordance with the consensus statement made by the ACCP and the Society of Thoracic Surgeons [44]. This statement recommended RFA as a treatment option for high-risk patients with stage I NSCLC with peripheral lesions less than 3 cm in size. This statement also noted that the limited ability of RFA to control primary tumors was responsible for the limited enthusiasm for its use in patients who are not candidates for SBRT or sublobar resection. However, the role of RFA in the treatment of early-stage NSCLC should ultimately be determined by the results of studies with high-quality evidence comparing RFA with other local therapies in the future.

To overcome the limited local efficacy of RFA, Dupuy et al. [45] suggested combination therapy with RFA and conventional radiation therapy. They performed RFA followed by conventional external beam radiation therapy in 24 patients with stage I NSCLC. For tumors with a mean size of 3.4 cm, the local progression rate was 8% (2/24 patients) at a mean follow-up of 27 months. Considering the high rate of local progression of stage I NSCLC with RFA alone, this result appears quite promising and encourages the use of such a combination therapy for patients who are not candidates for sublobar resection or SBRT.

Although the use of RFA as a primary therapy for early-stage NSCLC may be limited, we do recommend the use of RFA as a second salvage treatment option for NSCLC that recurs after primary therapy [46, 47]. Kodama et al. [46] treated 44 consecutive patients with recurrent NSCLC with RFA. During a mean follow-up period of 29 months, the 1-, 3-, and 5-year overall survival rates were 98%, 73%, and 56%, respectively. The 1- and 3-year recurrence-free survival rates were 77% and 41%, respectively. Independent significant prognostic factors were sex and tumor size. Schoellnast et al. [47] used RFA in 33 patients with 39 NSCLC tumors that recurred after surgery, chemotherapy, and/or radiation. The technical success rate was 97%, and the median survival time was 21 months after RFA. We suggest that RFA may also be a good treatment option for patients with metachronous lung cancer that develops after treatment of a previous cancer.

RFA is another option in addition to conventional therapies for the treatment of NSCLC. RFA may be suitable for patients with early-stage NSCLC and NSCLC recurrence after therapy, even if they are unsuitable for conventional therapies. It has some notable advantages: it is minimally invasive (can be performed percutaneously under local anesthesia), costs less than surgery [41], has an insignificant impact on pulmonary function [15, 16, 18, 20], may be applied regardless of any previous treatments, and may be repeated whenever necessary.

## 5. Recent Development of Technologies for RFA

RFA has only recently emerged as a treatment option for NSCLC, and the techniques are still improving. For example, a navigation system to improve the ease of the RFA procedure has been developed [48, 49]. Santos et al. [48] showed the feasibility of performing RFA using an electromagnetic navigation system to guide percutaneous electrode placement. By using CT images obtained immediately before RFA, this system can provide reconstructed “near” real-time CT images without scanning CT for “true” real-time images. Electrode placement can be guided precisely using reconstructed images without exposing the patient or the physician to radiation.

It has been suggested that the high local failure rate after RFA of large tumors is partly attributable to difficulty in obtaining an adequate ablation volume by geometric overlap of multiple ablation zones, which was described by Dodd et al. [50]. Banovac et al. [49] reported a computed pretreatment planning system that enabled volumetric sculpting of the ablation zone to cover the tumor and the desired margin with a minimum number of overlapping ablations. This system was incorporated into an electromagnetic navigation system, which may also allow computed planning of electrode placement.

Researchers in Japan have attempted RFA under bronchoscopy guidance rather than percutaneous CT guidance. They developed a new internally cooled electrode catheter that was suitable for the forceps channel of the bronchoscopy. After an animal experiment [51], they used the catheter for bronchoscopy-guided RFA before surgical resection in 10 patients with clinical stage IA NSCLC [52]. No complications, including pneumothorax, occurred. Surgical specimens were used to histologically confirm a certain volume of ablated area within the tumor. This study indicates that this procedure has the potential to become a therapeutic tool for inoperable patients with stage I NSCLC. One advantage of bronchoscopy guidance over the percutaneous route may be a decreased risk of pneumothorax.

In addition to the previously discussed new technologies, other ablative technologies are being developed, including microwave ablation, cryoablation, and irreversible electroporation. In contrast to RFA, which mainly relies on thermal conduction to kill tissues, microwave ablation has a much broader power field and therefore relies less on conduction into tissues. The heat-sink effect of blood flow is more pronounced within the zone of conductive rather than active heating. Therefore, ablation with a larger power field may not be influenced as much by the heat-sink effect that limits the ablation zone produced by RFA, yielding a more uniform ablation zone [53].

Cryoablation is used to treat inoperable stage I NSCLC. It is associated with a high local control rate (97%) and favorable survival (3-year overall survival rate: 88%) [54]. Cryoablation has some advantages: multiple applicators may be simultaneously used, reducing procedure time, especially for large tumors; procedural pain is less because of the analgesic effect of freezing; and grounding pads are not required, eliminating grounding pad injuries. Furthermore, irreversible electroporation, a new nonthermal ablation modality, is being investigated in the lung [55]. This technology utilizes pulses of direct current that last from microseconds to milliseconds. These pulses generate an electric field that causes nanoscale pores to form in cell membranes, leading to cell death [56]. Irreversible electroporation has exciting advantages over existing thermal ablation modalities: freedom from the heat-sink effect; preservation of larger airways (bronchi) and large blood vessels with regeneration of epithelium and endothelium, respectively; and rapid healing of the ablated tissue as quickly as within 3 weeks after treatment, which was confirmed in a pig lung model [55]. Such developing technologies will improve the clinical outcomes of ablation therapies.

## 6. Conclusion

RFA for early-stage NSCLC is usually performed using local anesthesia under CT guidance. Mortality is quite rare, but it can occur due to pulmonary events. RFA procedures frequently cause pneumothorax (up to 63% of cases), but pneumothorax requiring chest drainage occurs in less than 15% of the procedures. Other severe complications are rare. Local tumor progression after RFA of stage I NSCLC occurs in 31% to 42% of patients. The 1-, 2-, 3-, and 5-year overall survival rates after RFA of stage I NSCLC were 78% to 100%, 53% to 86%, 36% to 88%, and 25% to 61%, respectively. The median survival time ranged from 29 to 67 months. The 1-, 2-, and 3-year cancer-specific survival rates after RFA of stage I NSCLC were 89% to 100%, 92% to 93%, and 59% to 88%, respectively. There is a higher frequency of local failure after RFA than after sublobar resection and SBRT. Thus, we suggest that RFA may currently be reserved for patients with early-stage NSCLC who are unfit for sublobar resection or SBRT, although it is unclear how much this increased local failure impacts survival outcomes in old and high-risk patients. However, the role of RFA in the treatment of early-stage NSCLC should ultimately be determined by evidence from high-quality comparison studies in the future. Various technologies are being developed to improve the clinical outcomes of RFA for early-stage NSCLC.

## Figures and Tables

**Table 1:** Summary of studies reporting outcomes of RFA for NSCLC.

Author, year, and type of study	Reference Number	Center	Number of patients (tumors)	Patient age (y)	Number of patients or tumors according to cancer stage	Tumor size (cm)	Follow-up period (mo)	Toxicities	Local efficacy	Survival
^§^Lee et al., 2004	[10]	Chonbuk National University in South Korea	26 (27)	68*	IA/IB/II/III/IV: 1/9/1/7/8, respectively	5.6**	9*	Mortality (due to acute respiratory distress syndrome): 4%	Overall proportion of LTP: 73% Proportion of LTP for stage I: 40%	1-/2-year OS: 50%/32%, respectively, median OS: 7 mo, 1-/2-year OS for stage I: 100%/53%, respectively, and mean OS for stage I: 21 mo

Fernando et al., 2005	[11]	Pittsburgh Medical Center in US	18 (21)	75*	I/II/III/IV: 9/2/3/4, respectively	2.8*	14*	Mortality (due to pulmonary embolus): 6%, PTX requiring drainage: 39%, and pneumonia: 11%	Proportion of LTP: 38%	1-/2-year OS: 83%/83%, respectively, and mean OS: 21 mo

Pennathur et al., 2007	[12]	Pittsburgh Medical Center in US	19 (19)	78*	IA/IB: 11/8, respectively	2.6**	28* for alive patients	No mortality and PTX requiring drainage: 63%	Proportion of LTP: 42%	1-/2-year OS: 95%/68%, respectively

^§^Simon et al., 2007, retrospective	[13]	Brown University in US	75 (80)	NA	IA/IB: 56/19, respectively	3.0**	NA	Mortality (due to exacerbation of pulmonary fibrosis)	NA	1-/2-/3-/5-year OS: 78%/57%/36%/27%, respectively, median OS: 29 mo, and median OS for stages IA and IB: 30 mo and 25 mo, respectively (*P* = 0.58)

Hiraki et al., 2007, retrospective	[14]	Okayama University in Japan	20 (20)	76**	IA/IB: 14/6, respectively	2.4**	22*	No grade 3 or more toxicities, PTX requiring drainage: 4%, overall PTX: 57%, and pleural effusion: 17%	Proportion of LTP: 35% 1-/2-/3-year LTP: 28%/37%/37%, respectively	1-/2-/3-year OS and CSS: 90%/84%/74% and 100%/93%/83%, respectively, mean OS: 42 mo, and 1-/2-/3-year OS for stages IA and IB: 93%/93%/80% and 83%/67%/NA, respectively

^§^Lencioni et al., 2008, prospective	[15]	Multicenter in US, UK, Italy, Germany, and Australia	33 (38)	67*	IA/IB/recurrent NSCLC: 10/3/20, respectively	2.2**	NA	No mortality and PTX requiring drainage: 13%	Proportion of LTP: 13%	2-year OS/CSS for stage I: 75%/92%, respectively

Lanuti et al., 2009, retrospective	[16]	Massachusetts General Hospital in US	31 (34)	70*	IA/IB: 29/5, respectively	2.0**	17*	No mortality, PTX: 13%, chest tube placement: 8%, minor hemoptysis: 16%, hemothorax: 5%, pneumonia: 16%, pleural effusion: 21%, neuropathy: 3%, and bronchopleural fistula: 8%	Proportion of LTP: 32%	1-/2-/3-year OS and DFS: 85%/78%/47% and 82%/57%/39%, respectively, and median OS and DFS: 30 mo and 26 mo, respectively

Zemlyak et al., 2010, retrospective	[17]	Stony Brook University in US	12	74**	I: 12	NA	NA	No mortality, PTX: 58%, and hemoptysis: 8%	Proportion of LTP: 33%	3-year OS/CSS/DFS: 88%/88%/50%, respectively

Hiraki et al., 2011, retrospective	[18]	Okayama University in Japan	50 (52)	75**	IA/IB: 38/12, respectively	2.1**	37*	No grade 4 or 5 toxicities, grade 3 toxicities: 6% (including pleural effusion [2%], bronchopleural fistula [2%], or empyema [2%]), grade 2 toxicities: 12% (including PTX and/or pneumonitis), and grade 1 PTX: 42%	Proportion of LTP: 31%	1-/2-/3-/5-year OS, CSS, and DFS: 94%/86%/74%/61%, 100%/93%/80%/74%, and 82%/64%/53%/46%, respectively, median and mean OS: 67 mo and 59 mo, respectively, median and mean DFS: both 42 mo, and 1-/2-/3-/5-year OS for stages IA and IB: 95%/89%/83%/66% and 92%/75%/50%/50%, respectively (*P* = 0.057)

^§^Huang et al., 2011	[19]	Fourth Military Medical University in China	237	68*	I/II/III/IV: 33/50/109/45, respectively	NA	NA	NA	NA	1-/2-/5-year OS: 80%/46%/24%, respectively

Ambrogi et al., 2011, prospective	[20]	University of Pisa in Italy	57 (59)	74**	IA/IB: 44/15, respectively	2.6**	46*	No mortality and PTX requiring drainage: 5%, minor complications: 20% (including pain [6%], small PTX [6%], tiny pleural effusion [4%], minor hemoptysis [3%], and chest wall hematoma [1%])	Overall proportion of LTP: 41% Proportion of LTP for stage IA/IB: 34%/60%, respectively (*P* = 0.01)	1-/3-/5-year OS and CSS: 83%/40%/25% and 89%/59%/40%, respectively, median OS and CSS: 33 mo and 41 mo, respectively, median OS/CSS for stages IA and IB: 35 mo/52 mo and 20 mo/25 mo, respectively (OS and CSS significantly different between stages IA and IB), and 1-/3-/5-year OS for stage IA: 95%/71%/52%, respectively

Simon et al., 2012, retrospective	[21]	Brown University in US	82	76**	IA/IB/II/III/IV: 58/14/3/4/3, respectively	NA	16*	No mortality	NA	1-/2-/3-/5-year OS: 77%/62%/51%/21%, respectively, median OS: 37 mo, and 1-/3-year OS for stages IA and IB: 84%/76% and 79%/67%, respectively

Lanuti et al., 2012, prospective	[22]	Massachusetts General Hospital in US	45 (55)	70*	I: 45	2.0**	32*	Overall PTX: 18% and PTX requiring drainage: 2%	Proportion of LTP: 33%	3-/5-year OS: 67%/31%, respectively, and median OS: 44 mo

Lee et al., 2012, retrospective	[23]	Seoul Medical Center in South Korea	40	72**	I/II/II/IV: 15/1/13/11, respectively	3.8** for stages I and II and 4.6** for stages III and IV	56* for stages I and II and 37* for stages III and IV	Major complication: 15% (including pneumomediastinum [3%], hemothorax [3%], PTX [8%], and hemoptysis [3%])	Proportion of LTP: 40%	1-/2-/5-year OS for stages I and II: 100%/77%/19%, respectively, 3-year CSS for stages I and II: 33%, and median OS for stage I: 38 mo

*Median values, **mean values, and  ^§^the study is performed using a mixed population comprising both primary and metastatic lung cancer patients; data confined to NSCLC are extracted.

RFA = radiofrequency ablation, NSCLC = nonsmall cell lung cancer, NA = not available, PTX = pneumothorax, LTP = local tumor progression, OS = overall survival, CSS = cancer-specific survival, and DFS = disease-free survival.

**Table 2:** Summary of recent studies reporting outcomes of SBRT for stage I NSCLC.

Author, year, and type of study	Reference Number	Center	Number of patients (stage IA/IB)	Patient age (y)	Tumor size (cm)	Follow-up period (mo)	Toxicities	Local efficacy	Survival
Onishi et al., 2004	[24]	Yamanashi Medical University in Japan	35 (15/20)	78*	33**	13*	No grade ≥3 toxicities	Proportion of LTP: 6%	2-year OS and CSS: 58% and 83%, respectively

Nagata et al., 2005, prospective	[25]	Kyoto University in Japan	45 (32/13)	77* for stage IA and 73* for stage IB	<4.0	30* for stage IA and 22* for stage IB	No grade ≥3 pulmonary toxicities	Proportion of LTP: 2%	1-/2-/3-/5-year OS and DFS for stage IA: 93%/90%/83%/83% and 80%/72%/72%, respectively 1-/2-/3-/5-year OS and DFS for stage IB: 82%/72%/72%/72% and 92%/71%/71%/71%, respectively

Zimmermann et al., 2005	[26]	Technical University in Germany	30 (5/25)	60–69 (*n* = 10) 70–79 (*n* = 14) ≥80 (*n* = 6)	NA	18* for alive patients	No grade 5 or 4 toxicities and grade 3 pneumonitis: 3%	Proportion of LTP: 7% and 2-year LTP: 13%	1-/2-year OS: 80%/75%, respectively

Nyman et al., 2006	[27]	Sahlgrenska University in Sweden	45 (18/27)	74*	3.5**	43*	No grade 5 toxicities and no grade ≥2 radiation pneumonitis	Proportion of LTP: 20%	1-/2-/3-/5-year OS and CSS: 80%/71%/55%/30% and 88%/83%/67%/41%, respectively, and median OS and CSS: 39 mo and 55 mo, respectively

Timmerman et al., 2006, prospective	[28]	Indiana University in US	70 (35/35)	70*	NA	18*	Grade 5 toxicities: 9% (including pneumonia [6%], pericardial effusion [1%], hemoptysis [1%]) and grade 3 or 4 toxicities: 11%	Proportion of LTP: 4% and 2-year LTP: 5%	2-year OS: 55% and median OS: 33 mo

Onishi et al., 2007, retrospective	[29]	Multicenter in Japan	257 (164/93)	74*	2.8*	38*	No mortality and grade ≥3 pulmonary toxicities: 5%	Proportion of LTP: 14%,	3-/5-year OS and CSS: 57%/47% and 77%/73%, respectively

Baumann et al., 2009, prospective	[30]	Multicenter in Sweden, Norway, and Denmark	57 (40/17)	75*	2.5*	35*	No grade 5 toxicities and grade 4/3 toxicities: 2%/28%, respectively	Proportion of LTP: 7% and 3-year LTP: 8%	1-/2-/3-year OS and CSS: 85%/65%/60% and 93%/88%/88%, respectively, and median OS: 41 mo

Fakiris et al., 2009, prospective	[31]	Indiana University in US	70 (34/36)	NA	NA	50*	Grade 5 toxicities: 7% (including pneumonia [4%], hemoptysis [1%], and respiratory failure [1%]) and grade 4/3 toxicities: 1%/9%, respectively	Proportion of LTP: 6%, 3-year LTP: 12%	3-year OS/CSS: 43%/82%, respectively, median OS: 32 mo, and median OS for stages IA and IB: 39 mo and 25 mo, respectively

Ricardi et al., 2010, prospective	[32]	University of Torino in Italy	62 (43/19)	74*	2.4*	28*	No grade 5 toxicities and grade ≥3 radiation pneumonitis: 3%	Proportion of LTP: 6%, 3-y LTP: 12%	2-/3-year OS, CSS, and DFS: 69%/57%, 79%/73%, and 63%/55%, respectively

Timmerman et al., 2010, prospective	[33]	Multicenter in US and Canada	55 (44/11)	72*	≤5.0	34*	No grade 5 toxicities and grade 4/3 toxicities: 4%/13%, respectively	Proportion of LTP: 2%, 3-y LTP: 2%	3-year OS/DFS: 56%/48%, respectively, and median OS and DFS: 48 mo and 34 mo, respectively

Onishi et al., 2011, retrospective	[34]	Multicenter in Japan	87 (64/23)	74*	2.1* for stage IA and 3.9* for stage IB	55*	No grade 5 toxicities, grade 3 pulmonary toxicities: 1%, and overall grade 3 toxicities: 9%	Overall proportion of LTP: 9%, 5-year overall LTP: 13%, and 5-year LTP for stages IA and IB: 8% and 27%, respectively	5-year OS/CSS: 70%/76%, respectively, and 5-year OS for stages IA and IB: 72% and 63%, respectively

Lagerwaard et al., 2012, prospective	[35]	VU University Medical Center in Netherlands	177 (106/71)	76*	2.6*	32*	No grade 5 toxicities and grade ≥3 radiation pneumonitis: 2%	Proportion of LTP: 5% and 1-/3-year LTP: 2%/7%, respectively	1-/3-/5-year OS: 95%/85%/51%, respectively, and median OS: 62 mo

Shibamoto et al., 2012, prospective	[36]	Multicenter in Japan	180 (128/52)	77*	2.7*	36*	No grade 5 toxicities and grade 3 radiation pneumonitis: 1%	3-year LTP: 17%	3-/5-year OS: 69%/52%, respectively, and 3-year OS/CSS for stages IA and IB: 78%/88% and 60%/69%, respectively

Crabtree et al., 2014, retrospective	[37]	Washington University in US	151 (110/41)	74**	2.6**	23*	NA	Proportion of LTP: 11%	1-/3-/5-year OS and DFS: 82%/47%/25% and 79%/42%/19%, respectively

*Median values, **mean values, SBRT = stereotactic body radiation therapy, NSCLC = nonsmall cell lung cancer, NA = not available, LTP = local tumor progression, OS = overall survival, CSS = cancer-specific survival, and DFS = disease-free survival.
